# South West and North Central Nigeria: Assessment of cassava mosaic disease and field status of *African cassava mosaic virus* and *East African cassava mosaic virus*

**DOI:** 10.1111/aab.12647

**Published:** 2020-10-19

**Authors:** Angela O. Eni, Oghenevwairhe P. Efekemo, Olabode A. Onile‐ere, Justin S. Pita

**Affiliations:** ^1^ Department of Biological Sciences, College of Science and Technology Covenant University Ota Nigeria; ^2^ West African Virus Epidemiology (WAVE) for Root and Tuber Crops Ota Nigeria; ^3^ Laboratory of Plant Physiology Université Felix Houphouët‐Boigny (UFHB) Abidjan Côte d'Ivoire

**Keywords:** ACMV, *Bemisia tabaci*, CMD, EACMV, *Manihot esculenta* Crantz, Nigeria

## Abstract

Cassava mosaic disease (CMD), caused by cassava mosaic begomoviruses (CMBs), is a major threat to cassava production in Nigeria. The predominant CMBs in Nigeria are African cassava mosaic virus (ACMV), East African cassava mosaic virus (EACMV) and East African cassava mosaic Cameroon virus (EACMCV), which are transmitted through infected stem cuttings and whitefly vectors. This study was conducted in 2015 and 2017 to assess the epidemiology of CMD and the current distribution of CMBs in cassava farms in South West (SW) and North Central (NC) Nigeria. A survey of cassava farms was undertaken, and samples representative of disease symptoms were collected and assessed using molecular techniques. A total of 184 and 328 cassava farms were sampled in 2015 and 2017, respectively. CMD incidence for both regions surveyed was 43.80 and 12.25% in 2015 and 2017, respectively. Fields in SW recorded a higher incidence rate in 2015 (SW: 45.11%, NC: 42.47%), while the reverse occurred in 2017 (SW: 10.90%, NC: 14.01%). Overall, the CMD incidence in Benue State (NC) was significantly higher than other locations surveyed in both years. CMD symptom severity and mean whitefly population were higher in SW Nigeria in the two survey years. ACMV was widespread across both zones, occurring in 79.1% (453/613) and 54.8% (386/704) of cassava leaf samples analysed in 2015 and 2017, respectively. EACMV was detected in only 6.0% (37/613) and 4.7% (33/704) of all cassava leaf samples analysed in 2015 and 2017, respectively. Overall, a higher proportion of infected samples were found in NC in both 2015 (NC: 85.2%, SW: 75.4%) and 2017 (NC: 73.6%, SW: 45.2%). Detection using strain‐specific primers revealed that 97% of EACMV positive samples were indeed infected by the EACMCV strain of the virus. As previously reported, samples with mixed infections showed a higher symptom severity than samples with single ACMV or EACMV infections. This study provides an update to the distribution of CMBs in SW and NC Nigeria and will be useful in development of monitoring and management strategies for the disease in both regions.

## Graphical abstract

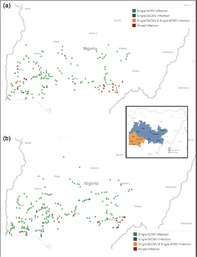

## 1 INTRODUCTION

Cassava, *Manihot esculenta* Crantz, is an important staple food that serves as an affordable source of carbohydrates for over 800 million people across Africa (FAO, 2013). In Nigeria, cassava provides more than half of the daily calorie requirement for people across various ethnic groups (Akinpelu, Amanigbo, Olojede, & Oyekale, 2011). It is considered an important food security crop, mainly because of the ease of production and processing, as well as its ready‐to‐eat product referred to as “garri” which can be stored for up to 12 months at room temperature (Akinpelu et al., 2011). Besides its importance for food, cassava is also an industrial raw material and thus a potential source of income to farmers and the country at large. Although Nigeria is the largest producer of cassava in Africa, with about 60 million tonnes produced annually (FAO, 2017), the five to 10 t/ha tuber yield common in Nigeria is much lower than the average tuber yield of 25 t/ha obtained in other cassava growing regions around the world (FAO, 2017). One major biotic constraint to cassava production is its susceptibility to cassava mosaic disease (CMD), a viral disease which causes annual tuber yield losses estimated at USD 1.9 to 2.7 billion (Patil & Fauquet, 2009).

CMD is caused by a group of viruses commonly referred to as cassava mosaic begomoviruses (CMBs), belonging to the genus *Begomovirus* in the family *Geminiviridae* (Ariyo, Koerbler, Dixon, Atiri, & Winter, 2005; Patil & Fauquet, 2009; Thottappilly, Thresh, Calvert, & Winter, 2003). The CMBs are characterised by their circular, bipartite single stranded DNA genome of about 2.7–2.9 kb (Kathurima, Ateka, Nyende, & Holton, 2016). The bipartite genome consists of two components, DNA‐A and DNA‐B (Haley, Zhan, Richardson, Head, & Morris, 1992). The CMBs are transmitted through use of infected cassava cuttings as planting materials and by whitefly vectors belonging to the *Bemisia tabaci* complex (Elfekih et al., 2018; Legg et al., 2015). Characteristic symptoms of CMD vary from mosaic patterns on cassava leaves to leaf distortion, vein clearing and stunted growth (Sseruwagi, Sserubombwe, Legg, Ndunguru, & Thresh, 2004). Several strains of CMBs have been identified to cause CMD in Africa namely *African cassava mosaic virus* (ACMV) *South African cassava mosaic virus* (SACMV), *East African cassava mosaic virus* (EACMV), *East African cassava mosaic Cameroon virus* (EACMCV), *East African cassava mosaic Zanzibar virus* (EACMZV), *East African cassava mosaic Malawi virus* (EACMMV), and *East African cassava mosaic Kenya virus* (EACMKV). Recently, two species have also been described: *African cassava mosaic Burkina Faso virus* (ACMBFV) and *Cassava mosaic Madagascar virus* (Harimalala et al., 2015; Tiendrébéogo et al., 2012).

Nationwide surveys conducted in Nigeria for the assessment of CMD and CMB in 2002 and 2003 revealed that ACMV, EACMV and EACMCV are the predominant CMBs responsible for CMD (Ariyo et al., 2005; Ogbe, Dixon, Hughes, Alabi, & Okechukwu, 2006). CMBs have also been identified in other plant species besides cassava including *Laportea* (*Fluerya*) *aestuans* (Urticaceae), *Senna occidentalis* (L.) Link, *Manihot glaziovii*, *Combretum confertum* (Benth.) M.A. Lawson, *Ricinus communis* L., *Leucana leucocephala* (Lam.) De Witt, *Glycine max* (L.) Merr. (Alabi et al., 2007, 2008; Mgbechi‐Ezeri, Alabi, Naidu, & Lava Kumar, 2008; Ogbe et al., 2006; Rossel, Thottappilly, Van Lent, & Huttinga, 1987). These non‐cassava CMBs hosts can serve as a reservoir for the spread of CMBs through whitefly transmission.

Cassava is grown in all agro‐ecological zones in Nigeria and in all 36 States of the Country as well as the Federal Capital Territory (FCT), Abuja. The 36 States of Nigeria of the country are grouped into six geopolitical zones (North Central [NC], North East, North West, South West [SW], South East and South–South), and cassava has various degrees of importance as food and feed in each zone. Approximately 50% of all cassava production in Nigeria takes place in the two of these six zones; NC and SW zones (FAO, 2004; Philips et al., 2005), making these two zones very important for cassava production in Nigeria. Given that the last CMD surveillance activities in these zones took place over ten years ago (Alabi et al., 2007, 2008; Ariyo et al., 2005; Ogbe et al., 2006), a comprehensive farm and diagnostic survey was undertaken over two years to assess the current status of CMD in these zones. The expected outcome of this survey is an update on CMD incidence, symptom severity, whitefly abundance as well as the distribution of ACMV, EACMV and EACMCV in these regions of Nigeria and this will also inform the development of future monitoring and management strategies for the disease.

## 2 MATERIALS AND METHODS

### 2.1 Survey area

**FIGURE 1:**
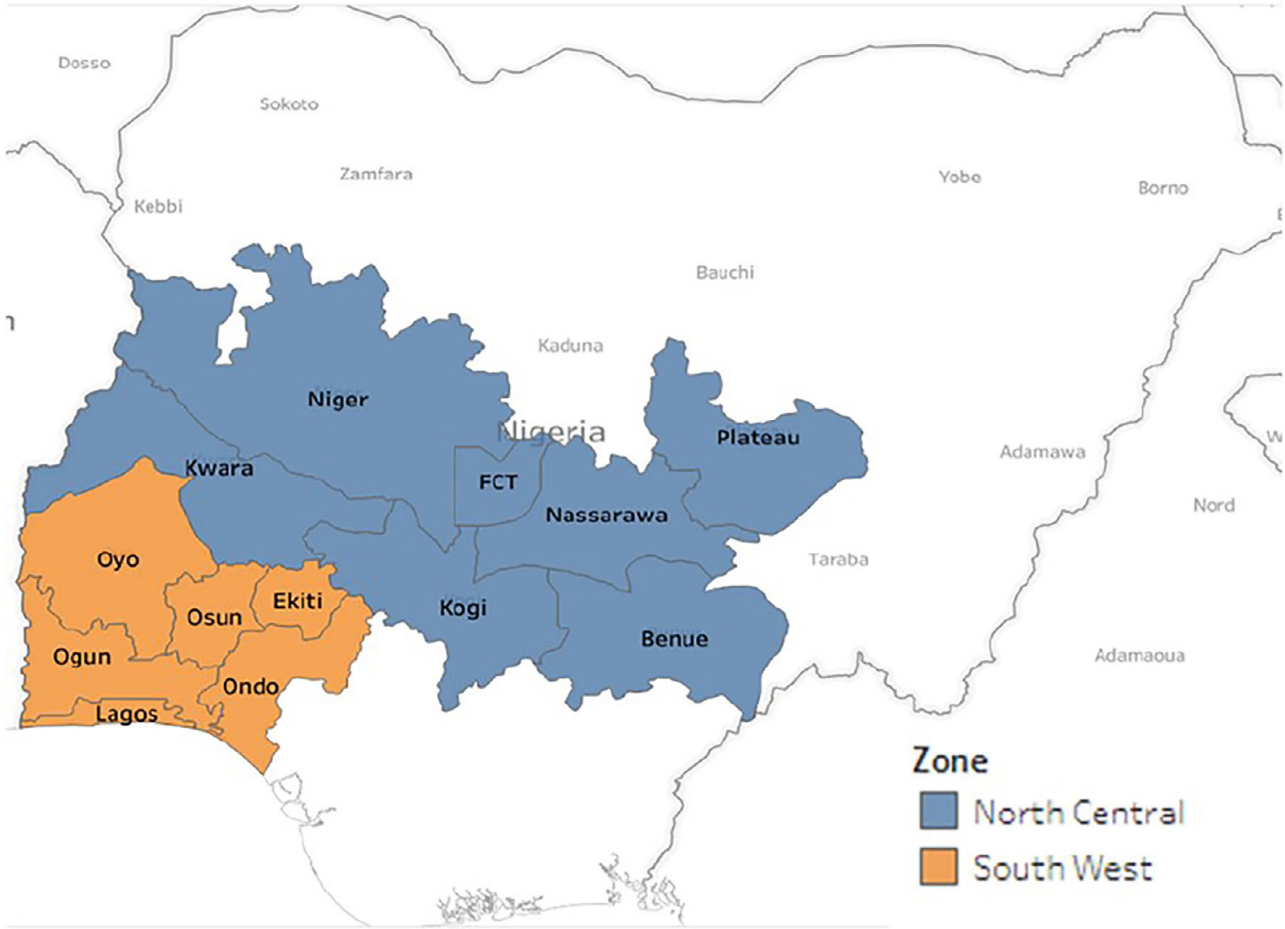
Map of Nigeria indicating the North Central and South West zones surveyed for cassava virus diseases in 2015 and 2017

A 2‐year survey (2015 and 2017) was conducted in two geopolitical zones of Nigeria: SW Zone (Oyo, Ogun, Ondo, Lagos, Ekiti and Osun States) and NC Zone (Nassarawa, Kogi, Plateau, Benue, Niger and Kwara States), including the FCT, Abuja (Figure 1).

A harmonised farm sampling protocol was adopted following a previously described method (Sseruwagi et al., 2004). Survey routes followed a road map which allowed sampling of cassava farms in various local government areas of the States. Surveyed cassava farms were a minimum of 10 km apart as described by Ogbe et al. (2006). The number of cassava farms between sample locations was recorded as a measure of the relative density of cassava, and the cassava varieties planted in each surveyed farm were also recorded. Geo‐location coordinates of farms were recorded using a GPS equipment (Garmin Inc., KS).

### 2.2 Incidence and symptom severity assessment

In each farm sampled, 30 cassava plants were assessed randomly for the presence or absence of CMD symptoms along two diagonals. The CMD incidence was calculated as the percentage of CMD‐symptomatic plants in relation to the number of plants assessed. For each cassava plant, CMD symptom severity was scored on a scale of 1 to 5:1 = asymptomatic plants, 2 = plants with 25% of leaves showing mild chlorotic pattern or mild distortion, 3 = infected plants with 50% exhibiting moderate mosaic pattern, narrowing and distortion at base of the leaves, 4 = infected plants with 75% exhibiting severe mosaic symptom, leaf distortion and general reduction of leaf size, and 5 = infected plants with 100% of plants exhibiting severe mosaic, leaf distortion, reduced leaf size, vein clearing and in most cases stunted growth (Sseruwagi et al., 2004). At each farm, a minimum of one and a maximum of four leaf samples were collected from asymptomatic and symptomatic cassava plants of varying disease severity. Leaf samples from weeds showing mosaic symptoms were also collected for assessment of their role as possible alternative host plants for ACMV or EACMV. All samples were stored in herbarium presses prior to laboratory analysis.

### 2.3 Source of CMD infection and whitefly assessment

The possible source of the observed CMD infection in each plant was determined based on the location of the leaf symptoms. Cassava plants that showed symptoms either only on the lower leaves or on all leaves were assumed to have been infected through the use of infected cassava cuttings. Plants that showed symptoms only on their upper leaves but not on any lower leaves were assumed to have been infected by the whitefly vector (Sseruwagi et al., 2004). The whitefly population on each assessed plant was estimated by counting the number of whiteflies on the three topmost leaves (Fargette, Fauquet, & Thouvenel, 1985; Samura et al., 2014).

### 2.4 DNA extraction

Total DNA was extracted from cassava leaf and weed samples following the protocol of Dellaporta, Wood, and Hicks (1983). The DNA pellet was reconstituted in 50 μL of TE buffer and stored at −20°C until further use. The concentration of each isolated DNA was determined using a NanoDrop 2000 spectrophotometer (Thermo Fisher Scientific, Waltham, MA) and adjusted to 50 ng/μL for use in PCR.

### 2.5 Polymerase chain reaction

PCR was performed using specific primers (Table 1) for the detection of ACMV, EACMV and EACMCV in the leaf samples collected during the 2‐year survey. DNA template from previously characterised isolates was used as controls for the PCR. Each reaction mixture consisted of 10× PCR reaction buffer (200 mM Tris HCl [pH 8.4], 500 mM KCl), 10 mM dNTPs (Promega), 25 mM MgCl_2_, 20 pmole of each primer, and 1 U Taq DNA Polymerase (Promega). The amplification conditions were as follows: an initial denaturation at 94°C for 2 min followed by 30 cycles of denaturation at 94°C for 1 min, annealing at 55°C for 1 min, extension at 72°C for 1 min and a final extension of 72°C for 10 min. Following amplification, PCR products were separated by electrophoresis alongside a 1 kbp plus DNA ladder (Thermo Fisher Scientific) on a 1% agarose gel stained with ethidium bromide (10 mg/ml). The gel was viewed under UV light using a Bench top UV transilluminator (UVP).

**TABLE 1:** Primer pairs used for the amplification of *African cassava mosaic virus* (ACMV), *East African cassava mosaic virus* (EACMV) and the *East African cassava mosaic Cameroon virus* (EACMCV)

Primer name	Primer sequences (5′ to 3′)	Target region	Size	Reference
JSP 001	ATGTCGAAGCGACCAGGAGAT			
JSP 002	TGTTTATTAATTGCCAATACT	ACMV DNA‐A (CP)	783 bp	Pita et al., 2001
ACMVBF	TCGGGAGTGATACATGCGAAGGC			
ACMVBR	GGCTACACCAGCTACCTGAAGCT	ACMV DNA‐B (BV1/BC1)	628 bp	Matic, Pais da Cunha, Thompson, & Tepfer, 2012
JSP 001	ATGTCGAAGCGACCAGGAGAT			
JSP 003	CCTTTATTAATTTGTCACTGC	EACMV DNA‐A (CP)	780 bp	Pita et al., 2001
VNF031/F	GGATACAGATAGGGTTCCCAC			
VNF032/R	GACGAGGACAAGAATTCCAAT	EACMV‐CM DNA‐A (AC2/AC3)	≈ 560 bp	Fondong et al., 2000
EAB555/F	TACATCGGCCTTTGAGTCGCATGG			
EAB555/R	CTTATTAACGCCTATATAAACACC	EACMV DNA‐B (BC1/CR)	544–560 bp	Fondong et al., 2000
EACMV1	GTTCGGCTATCACCTTCTAGAACA			
EACMV2	CAAGGCTTACATTGAAAAGGGA	EACMV‐BC1 (DNA‐A)	375 bp	Matic et al., 2012

### 2.6 Analysis of field data

Descriptive statistics were used to describe distributions. Continuous dependent variables, such as CMD incidence, CMD severity, percentage of cutting infection and percentage of whitefly infection were checked for normality using the Shapiro–Wilk and Kolmogorov–Smirnov tests alongside a histogram. Kruskal–Wallis test was performed to assess the difference in the distribution of dependent variables across States and years. Pairwise tests were also performed for post hoc Kruskal–Wallis comparisons. Spearman's correlation was used to examine the relationship between continuous variables. Significance was considered to be *p* < .05 for all tests. Distribution maps were generated using the GPS data alongside CMD distribution information. All statistical analyses were performed using SPSS v20 for Windows and maps were generated using Tableau 10.5.

## 3 RESULTS

### 3.1 Assessment of CMD symptoms

**FIGURE 2:**
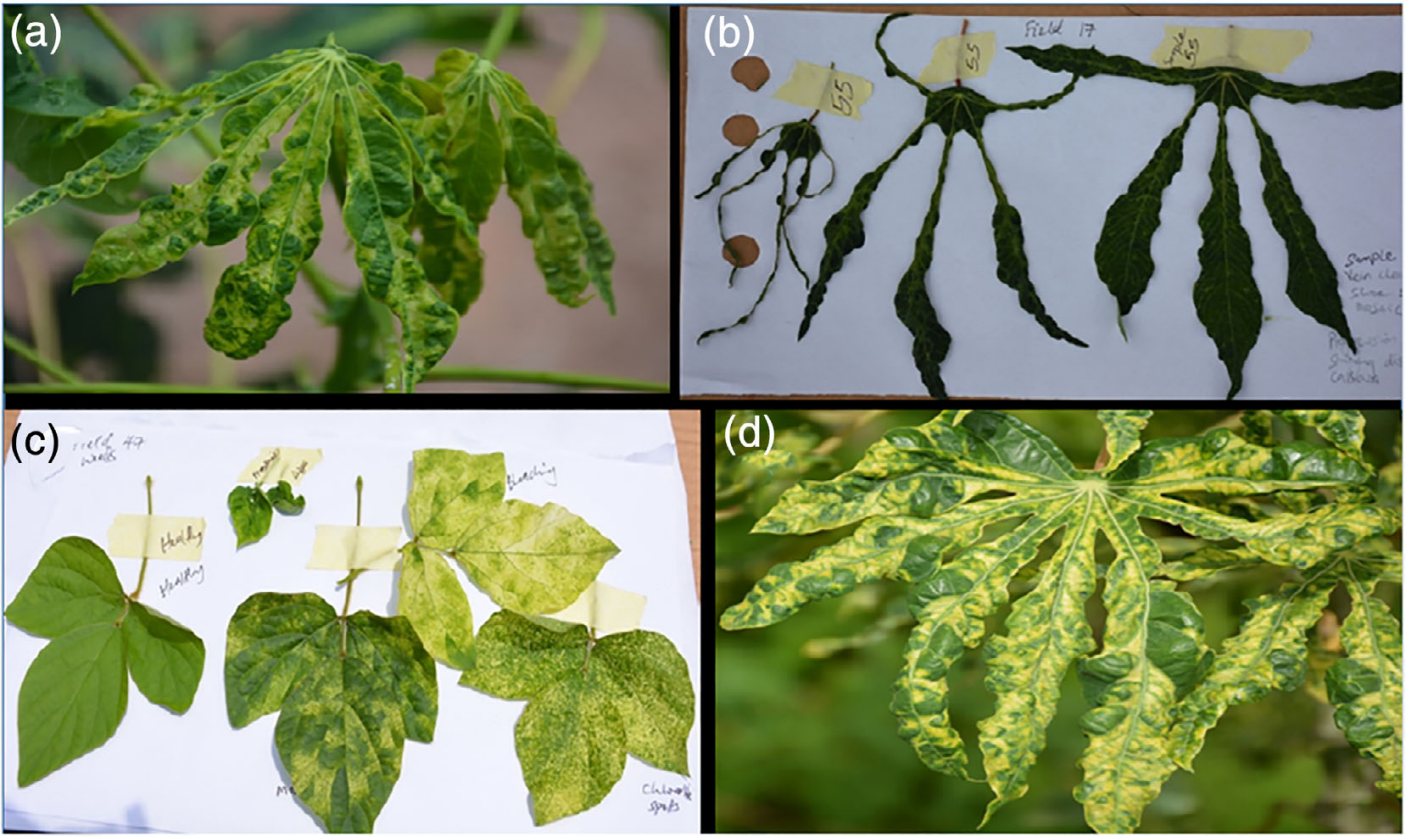
Virus symptoms observed in cassava farms in North Central and South West zones of Nigeria: (a) Green mosaic. (b) Shoe stringing. (c) Various mosaic patterns observed in a Fabaceae weed within a cassava farm. (d) Yellow mosaic

Symptoms of CMD including distinctive leaf mosaic and distortion were observed on cassava plants as well as on potential alternative host plants (weeds) across all States in the NC and SW. Symptomatic weed samples collected during this study included *Centrosema pubescens* Benth., *Chromolaena odorata* (L.) King and Robinson, *Senna alata* (Linn.) Roxb. and some unidentified weeds of the Cucurbitaceae and Fabaceae families. Other viral disease symptoms observed included vein clearing, leaf puckering and stunted growth of cassava plants (Figure 2).

### 3.2 CMD incidence and symptom severity

Visual assessment of symptoms showed an overall CMD incidence of 43.80 and 12.25% in 2015 and 2017, respectively (Table 2). In 2015, CMD incidence was higher in the SW (45.11%) but the NC had a higher incidence in 2017 (14.01%). The differences in CMD incidence between the two Zones in 2015 and 2017 were, however, not statistically significant (2015: *p* = .559, 2017: *p =* .661). Among the SW States, CMD incidence was highest in Ogun (49.76%) and Lagos (20.74%) States in 2015 and 2017, respectively. Benue State had the highest CMD incidence among the NC States in both years and also had the highest incidence of all States surveyed in the 2 years of the study (Table 2). Plateau had the lowest CMD incidence in the NC States for both years, while Lagos and Osun recorded the lowest incidence among SW States in 2015 and 2017, respectively (Table 2).

**TABLE 2:** Mean incidence and severity of CMD in cassava farms surveyed in South West and North Central Nigeria in 2015 and 2017

Zone	State	2015				2017			
		No. of farms	No. of laboratory tested samples	Mean CMD incidence (%)	Mean symptom severity	No. of farms	No. of laboratory tested samples	Mean CMD incidence (%)	Mean symptom severity
North Central	Abuja	1	3	100.00	2.27	2	4	16.67	2.00
	Benue	30	114	59.11	2.69	34	68	26.37	2.10
	Kogi	16	38	29.17	2.50	32	51	6.67	2.09
	Kwara	12	43	49.87	2.59	20	36	8.17	2.28
	Nassarawa	10	31	30.33	2.93	30	48	11.89	2.05
	Niger	13	39	38.46	2.96	13	26	20.00	2.12
	Plateau	9	15	13.70	3.11	11	18	6.06	2.31
	Subtotal	91	283	42.47^a^	2.72^a^	142	251	14.01^b^	2.11^b^
South West	Ekiti	11	45	46.06	3.13	20	33	10.33	2.01
	Lagos	3	9	23.33	2.60	9	24	20.74	2.16
	Ogun	28	104	49.76	2.67	36	103	18.15	2.13
	Ondo	15	68	41.78	2.74	39	89	8.46	2.12
	Osun	12	47	37.78	2.80	32	78	6.04	2.22
	Oyo	24	80	47.71	2.65	50	130	9.13	2.19
	Subtotal	93	353	45.11 ^a^	2.75 ^a^	186	457	10.90 ^b^	2.14 ^b^
Total		184	636	43.80 ^a^	2.73 ^a^	328	708	12.25 ^b^	2.13 ^b^

*Note*: Values with alphabetical superscripts across years signify a significant difference (*p* < .05) in mean cassava mosaic disease (CMD) incidence and mean symptom severity between both years.

Symptom severity was moderate in both years. The mean symptom severity was higher in 2015 (2.73) than in 2017 (2.13). The SW Zone had higher symptom severity scores in both years compared to the NC Zone (Table 2). However, this difference between the two Zones was not statistically significant in either year (2015: *p* = .735, 2017: *p =* .634). The CMD symptom severity was positively correlated with CMD incidence in 2015 (*r* = .231, *p* = .003) and 2017 (*r* = .373, *p* < .0001).

### 3.3 Origin of infection and adult whitefly distribution

**FIGURE 3:**
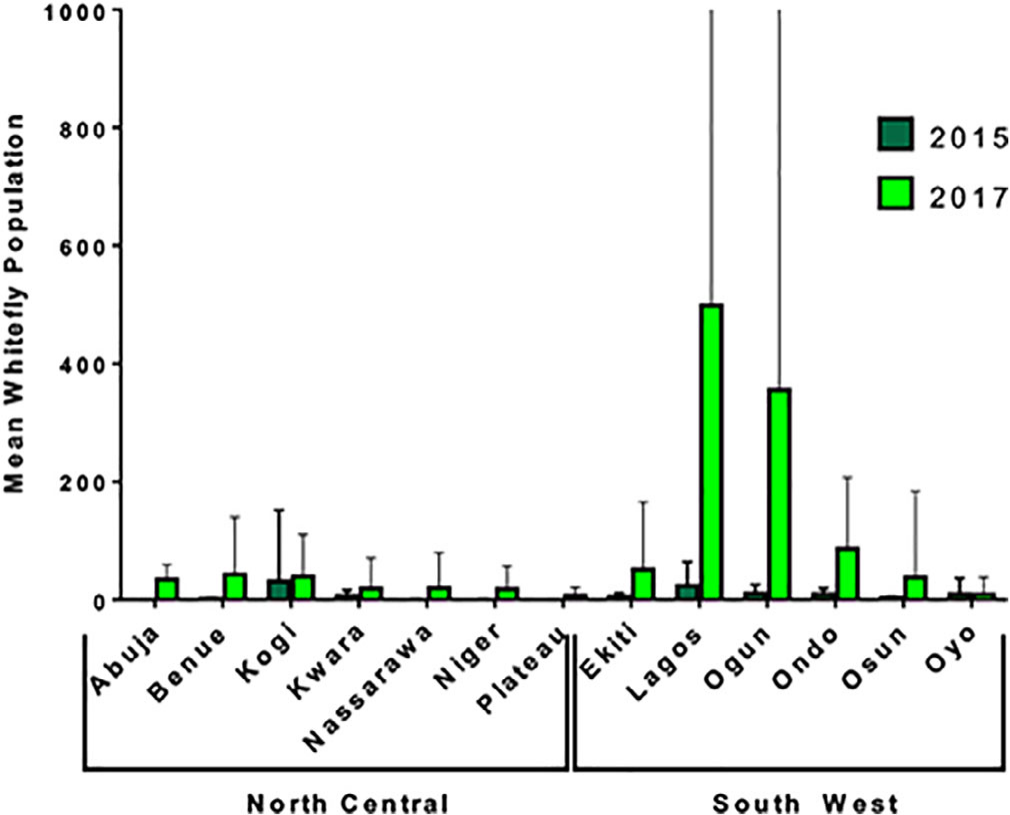
Mean whitefly population in 512 (2015 = 184; 2017 = 328) cassava farms sampled in South West and North Central Nigeria in 2015 and 2017. Error bar = *SD*

Based on the location of symptoms on sampled plants, we observed that CMD transmission in the surveyed zones were mostly as a result of the propagation of infected cassava cuttings (2015 = 79%; 2017 = 87%) than by transmission by whitefly vectors (2015 = 21%; 2017 = 13%). Average whitefly populations were higher in 2017 than in 2015 in both Zones and in all States (Figure 3). It was also observed that average whitefly populations were higher in the SW than the NC Zone in both years and were poorly correlated with CMD incidence in 2015 (*r* = .070, *p* = .342) and 2017 (*r* = .169, *p* = .002).

### 3.4 PCR detection of ACMV, EACMV and EACMCV in cassava leaf samples

A total of 613 and 704 cassava leaf samples were collected and analysed in 2015 and 2017, respectively. ACMV was widespread across both Zones having been found in 79.1% (485/613) and 54.8% (386/704) of all cassava leaf samples collected in 2015 and 2017, respectively. Proportion of ACMV positive samples was highest in the NC Zone in both survey years (Table 3). In 2015, the highest proportion of ACMV positive samples was found in Kogi (94.7%) and Plateau (93.3%) States in the NC Zone while Ogun (80.3%) and Ondo (70.9%) States had the highest proportions of ACMV positive samples in the SW Zone. The trend changed slightly in 2017 with the highest proportion of ACMV positive samples found in Niger (88.06%), Plateau (88.98%) States in the NC Zone and Lagos (66.7%), Ogun (66.0%) States in the SW Zone (Tables 4 and 5).

**TABLE 3:** Distribution of CMBs and type of infection across zones in both 2015 and 2017

Year	Zone	ACMV Single^a^	EACMV Single^b^	Mixed^c^	Negative^d^	Total
2015	North Central	219 (77.4%)	2 (0.7%)	20 (7.1%)	42 (14.8%)	283 (100.0%)
	South West	234 (70.9%)	3 (0.9%)	12 (3.6%)	81 (24.5%)	330 (100.0%)
	Total	453 (73.9%)	5 (0.8%)	32 (5.2%)	123 (20.1%)	613 (100.0%)
2017	North Central	168 (67.2%)	0 (0.0%)	16 (6.4%)	66 (26.4%)	250 (100.0%)
	South West	188 (41.4%)	3 (0.7%)	14 (3.1%)	249 (54.8%)	454 (100.0%)
	Total	356 (50.6%)	3 (0.4%)	30 (4.3%)	315 (44.7%)	704 (100.0%)

Abbreviations: ACMV, African cassava mosaic virus; CMB, cassava mosaic begomovirus; EACMV, East African cassava mosaic virus.

^a^ ACMV single infection.

^b^ EACMV single infection.

^c^ ACMV + EACMV mixed infection.

^d^ Negative for both ACMV and EACMV.

**TABLE 4:** Distribution of CMBs and type of infection across States in North Central and South West Nigeria in 2015

Zone	State	ACMV Single^a^		EACMV Single^b^		Mixed^c^		Negative^d^	
North Central	Abuja	3	100.0%	0	0.0%	0	0.0%	0	0.0%
	Benue	81	71.1%	2	1.8%	18	15.8%	13	11.4%
	Kogi	36	94.7%	0	0.0%	1	2.6%	1	2.6%
	Kwara	25	58.1%	0	0.0%	0	0.0%	18	41.9%
	Nassarawa	27	87.1%	0	0.0%	0	0.0%	4	12.9%
	Niger	33	84.6%	0	0.0%	1	2.6%	5	12.8%
	Plateau	14	93.3%	0	0.0%	0	0.0%	1	6.7%
	Total	219	77.4%	2	0.7%	20	7.1%	42	14.8%
South West	Ekiti	30	69.8%	0	0.0%	0	0.0%	13	30.2%
	Lagos	9	100.0%	0	0.0%	0	0.0%	0	0.0%
	Ogun	80	78.4%	0	0.0%	3	2.9%	19	18.6%
	Ondo	39	70.9%	1	1.8%	0	0.0%	15	27.3%
	Osun	30	68.2%	0	0.0%	0	0.0%	14	31.8%
	Oyo	46	59.7%	2	2.6%	9	11.7%	20	26.0%
	Total	234	70.9%	3	0.9%	12	3.6%	81	24.5%

Abbreviations: ACMV, African cassava mosaic virus; CMB, cassava mosaic begomovirus; EACMV, East African cassava mosaic virus.

^a^ ACMV single infection.

^b^ EACMV single infection.

^c^ ACMV + EACMV mixed infection.

^d^ Negative for both ACMV and EACMV.

**TABLE 5:** Distribution of CMBs and type of infection across States in North Central and South West Nigeria in 2017

Zone	State	ACMV Single^a^		EACMV Single^b^		Mixed^c^		Negative^d^	
North Central	Abuja	3	75.0%	0	0.0%	0	0.0%	1	25.0%
	Benue	46	67.6%	0	0.0%	12	17.6%	10	14.7%
	Kogi	30	58.8%	0	0.0%	3	5.9%	18	35.3%
	Kwara	19	52.8%	0	0.0%	0	0.0%	17	47.2%
	Nassarawa	32	66.7%	0	0.0%	1	2.1%	15	31.3%
	Niger	22	88.0%	0	0.0%	0	0.0%	3	12.0%
	Plateau	16	88.9%	0	0.0%	0	0.0%	2	11.1%
	Total	168	67.2%	0	0.0%	16	6.4%	66	26.4%
South West	Ekiti	15	46.9%	0	0.0%	1	3.1%	16	50.0%
	Lagos	16	66.7%	0	0.0%	3	12.5%	5	20.8%
	Ogun	68	66.0%	1	1.0%	5	4.9%	29	28.2%
	Ondo	34	38.2%	2	2.2%	3	3.4%	50	56.2%
	Osun	23	30.3%	0	0.0%	2	2.6%	51	67.1%
	Oyo	32	24.6%	0	0.0%	0	0.0%	98	75.4%
	Total	188	41.4%	3	0.7%	14	3.1%	249	54.8%

Abbreviations: ACMV, African cassava mosaic virus; CMB, cassava mosaic begomovirus; EACMV, East African cassava mosaic virus.

^a^ ACMV single infection.

^b^ EACMV single infection.

^c^ ACMV+EACMV mixed infection.

^d^ Negative for both ACMV and EACMV.

EACMV was detected at significantly lower rates than ACMV in both Zones and in both years. EACMV was detected in only 6.0% (37/613) and 4.7% (33/704) of all cassava leaf samples analysed in 2015 and 2017, respectively. EACMV was mostly detected in the NC Zone with Benue State having the highest proportion of positive samples in 2015 (17.6%) and 2017 (17.6%). In the SW Zone, EACMV was detected in 4.5 and 4.7% of samples collected in 2015 and 2017, respectively. Proportion of EACMV positive samples was highest in Oyo state (14.3%) in 2015 and Lagos state (12.5%) in 2017 (Tables 4 and 5). Most of the EACMV detected in cassava 97.3% (36/37) and 100% (33/33) in 2015 and 2017, respectively, were confirmed to be EACMCV using primers specific for EACMCV.

### 3.5 Single, mixed infections and symptom severity

Most CMB infections detected in cassava plants in this study were single ACMV infections in both years (2015: 73.9% and 2017: 50.6%) while only a small percentage of samples were singly infected by EACMV (2015: 0.8% and 2017: 0.4%) because EACMV occurred primarily as mixed infections with ACMV (Tables 4 and 5). Although the differences in symptom severity scores between the different infection types (Single ACMV infection, single EACMV infection or mixed infection of ACMV and EACMV) were not statistically significant (*p* > .05), symptom severity varied by type of infection. Samples with mixed infection of ACMV and EACMCV were observed to have a higher symptom severity scores and samples infected with ACMV alone had the lowest symptom severity scores out of all infection types (Table 6).

**TABLE 6:** Mean symptom severity score among various CMB infection type observed in 2015 and 2017

	2015		2017	
	Mean	*SD*	Mean	*SD*
ACMV Alone	3.16	0.93	2.38	0.59
EACMV Alone	3.33	1.15	3.00	0.0
EACMCV	3.46	1.04	2.42	0.72
Mixed	3.50	1.03	2.39	0.72

Abbreviations: ACMV, African cassava mosaic virus; CMB, cassava mosaic begomovirus; EACMCV, East African cassava mosaic Cameroon virus; EACMV, East African cassava mosaic virus.

### 3.6 Correlation between the presence of observable CMD symptoms and the presence of a virus in cassava plants

A total of 613 (symptomatic samples = 446; asymptomatic samples = 167) and 704 (symptomatic samples = 271; asymptomatic samples = 433) cassava leaf samples were collected in 2015 and 2017, respectively, for laboratory analysis. Almost half of all asymptomatic samples collected in 2015 and 2017 were positive for either ACMV or EACMV by PCR. Proportion of positive asymptomatic samples in 2015 included 38.9% (65/167), 1.2% (2/167) and 3.6% (6/167) for single ACMV, single EACMV and mixed infection, respectively. In 2017, proportion of positive asymptomatic samples included 33.7% (146/433), 0.5% (2/433) and 1.6% (7/433) for single ACMV, single EACMV and mixed infection, respectively. On the other hand, 6.5% and 13.7% of samples having observable symptoms tested negative for ACMV or EACMV in 2015 and 2017, respectively. Chi‐square analysis showed that single ACMV infections were significantly associated with the presence of an observable symptom in 2015 and 2017 (*p* < .05).

### 3.7 Cassava cultivars

Information on cassava variety was limited during the 2015 survey as such it is not reported here. During the 2017 survey, the type(s) of cassava variety planted in the surveyed fields were documented based on common names provided by the farmers. This data was collected for 92% of the fields surveyed in 2017 but were not recorded where the farmers were unavailable in the farm during the survey and/or where the variety is not one of the common ones. Most farms planted one cassava variety, but in some cases, more than one variety was present in a farm. We recorded only the major cassava variety in each farm. A total of 12 cassava varieties were recorded. Over 75% of the varieties recorded were local varieties with “*Akpu*” and “*Okowayo*” as the predominant varieties recorded in both Zones (Table 7). There were two improved varieties as reported by the farmers; the “*Agric*” and the “*TME 419*.” CMD incidence varied by variety with fields cultivating the Banada variety having the highest CMD incidence and highest proportion of cutting borne infections (Table 8). Symptom severity was generally low in the survey region and as such did not vary significantly by cultivar. Local varieties, however, showed more severe symptoms than the improved varieties. Whitefly abundance varied with fields planting “*Agric*,” “*Banada*” and “*Akpu*” having higher whitefly populations than other fields (Table 8). The observed higher whitefly population, however, was not correlated with a high proportion of whitefly borne infections on fields planting these varieties.

**TABLE 7:** Cassava cultivars observed in cassava farms in North Central and South West Nigeria in 2017 and the number of fields in which they were found

Cassava cultivar	North Central	South West
Agric	1	46
Ajasa	1	0
Akpu	36	115
Baki itche (black stem)	17	0
Banada	4	0
Cotonou	1	0
Danwari	11	0
Feri itche (white stem)	5	0
Odongbo	2	14
Okoyawo	45	53
Pinky	0	3
TME419	2	0

**TABLE 8:** Mean CMD incidence, CMD symptom severity, type of infection and whitefly abundance across fields where different cultivars were cultivated

Cassava cultivar	Number of fields	Mean CMD incidence	Mean symptom severity	Cutting infection (%)	Whitefly infection (%)	Mean whitefly abundance
Agric	47	8.51	2.09	51.41	16.77	207
Ajasa	1	0				0
Akpu	151	13.65	2.17	55.18	16.47	114
Baki itche (black stem)	17	7.47	2.10	36.35	4.83	27
Banada	4	33.25	2.073	86.723	13.28	152
Cotonou	1	0				65
Danwari	11	17.55	2.246	78.79	12.12	59
Feri itche (white stem)	5	13.8	2.143	28.58	31.42	14
Odongbo	16	21.88	2.24	49.16	25.84	21
Okoyawo	98	8.54	2.11	36.10	13.90	87
Pinky	3	28	2.12	76.40	23.6	0
TME419	2		0			27

*Note*: All values are averaged across all fields cultivating each cassava cultivar. Cutting infection and whitefly infection implies the average proportion of plants, across all fields cultivating each cultivar, with infections that originated from the propagation of an infected cutting or from the whitelfy vector.

**FIGURE 4:**
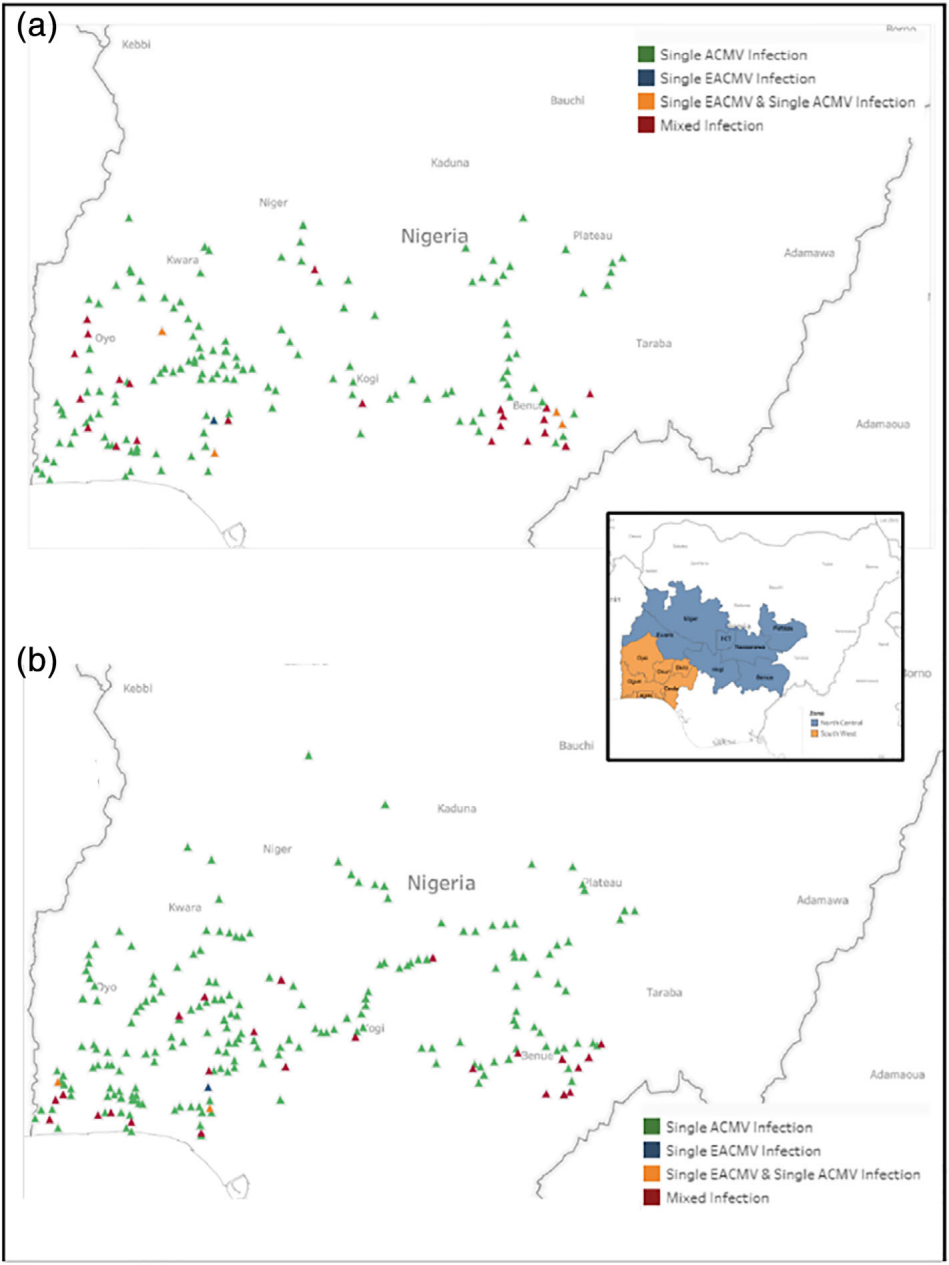
Distribution of single ACMV, single EACMV; single EAMCV and single ACMV; mixed infection (ACMV + EACMV) in South West and North Central Zones of Nigeria. (a) 2015. (b) 2017

The distribution of ACMV and EACMV varied across the various varieties (Figure 4). The highest proportion of ACMV and EACMV infected samples were collected from fields cultivating Danwari (75.76%), Baki itche (68.63%) and Banada (62.5%). Fields planting Danwari also had the highest proportion of samples with mixed ACMV and EACMV infections while fields cultivating Odongbo and Pinky had the lowest proportion of virus infected samples (Table 9).

**TABLE 9:** Proportion of ACMV, EACMV and mixed (ACMV + EACMV) infections in samples from fields planting each cultivar

Cassava cultivar	Total number of samples	ACMV (%)	EACMV (%)	Mixed (%)	Negative (%)
Agric	105	38.69	1.06	2.73	57.52
Ajasa	1	100	0	0	0
Akpu	358	46.81	0.77	4.38	48.04
Baki itche (black stem)	28	68.63	0	0	31.37
Banada	8	62.5	0	0	37.5
Cotonou	1	100	0	0	0
Danwari	22	62.12	0	13.64	24.24
Feri itche (white stem)	9	100	0	0	0
Odongbo	54	34.37	0	1.56	64.06
Okoyawo	185	40.05	0.34	2.89	56.72
Pinky	11	36.11	0	0	63.89
TME419	2	50	0	0	50

Abbreviations: ACMV, African cassava mosaic virus; EACMV, East African cassava mosaic virus.

### 3.8 Alternate hosts

A total of 27 (2015 = 23; 2017 = 4) alternate host samples (which included *Centrosema pubescens* Benth., *Chromolaena odorata* (L.) King and Robinson, *Senna alata* (Linn.) Roxb. and some unidentified weeds of the Cucurbitaceae and Fabaceae families) were collected and tested. Single ACMV and single EACMV were the most common infection types among alternate host samples. Proportion of positive alternate host samples in 2015 included 17.4% (4/23), 13.0% (3/23) and 4.4% (1/23) for single ACMV, single EACMV and mixed infection, respectively. All EACMV positive samples observed in alternate host samples were EACMCV infections. In 2017, only single ACMV infection was detected in 50% (2/4) of the alternate hosts samples analysed.

## 4 DISCUSSION

This study shows the presence of CMD in surveyed cassava farms with incidence of <50% in both years and symptom severity varying from mild to very severe as was reported in previous CMD surveys in Nigeria (Ogbe et al., 2006). Findings from this study, however, showed no correlation between whitefly population and CMD incidence as earlier reported in previous studies (Boykin et al., 2018; Manani, Ateka, Nyanjom, & Boykin, 2017; Toualy, Atta Diallo, Akinbade, Ska, & Lava Kumar, 2014). Studies conducted by the cassava diagnostics project in East Africa showed that the whitefly population is high in regions with whitefly‐susceptible cassava varieties and in regions with whitefly biotypes of high fecundity (Boykin et al., 2018; Dinsdale, Cook, Riginos, Buckley, & De Barro, 2010; Mugerwa, Rey, Tairo, Ndunguru, & Sseruwagi, 2019; Omongo et al., 2012). In addition, whitefly transmission of CMD is biotype dependent because studies have shown that certain whitefly biotypes are attracted by the yellow mosaic patterns of CMD‐infected cassava plants (Colvin et al., 2006; Manani et al., 2017; Omongo et al., 2012). This implies that when evaluating possible CMD management options like introduction of resistant varieties, the susceptibility of resistant varieties to various whitefly biotypes must be considered (Boykin et al., 2018; Dinsdale et al., 2010; Maruthi, Colvin, & Seal, 2001).

Another important factor to consider when introducing disease resistant varieties are the agronomic traits preferred by farmers. As observed in this study, over 75% of the farmers planted local varieties which points to the low adoption rate of improved varieties. Previous studies have shown that farmers are most likely to select varieties based on agronomic traits such as tuber yield, early maturation, durability in the soil and drought resistance (Afolami, Obayelu, & Vaughan, 2015; Bentley et al., 2017). The two predominant varieties observed in this study are both high yielding and have early maturation time. It is therefore important for breeders to consider farmer preferences in a bid to increasing the rate of adoption of resistant varieties.

Besides the use of improved varieties as a CMD management strategy, other strategies include the use of virus‐free planting materials. This study recorded a high percentage of CMD cutting transmitted infection as compared to whitefly transmitted CMD observed which is similar to previous studies conducted in Nigeria (Ogbe et al., 2006), Zambia (Chikoti et al., 2013), Rwanda (Night et al., 2011), Sierra Leone (Samura et al., 2014), Kenya (Mwatuni, Ateka, Karanja, Mwaura, & Obare, 2015) and the Central African Republic (Zinga et al., 2013). This implies that farmers are either unaware of the need for use of virus‐free planting materials or have limited access to virus‐free planting materials. In the absence of virus‐free planting materials, farmers can be trained on how to recognise CMD symptoms and select healthy cuttings for the next planting season (Mallowa, Isutsa, Kamau, Obonyo, & Legg, 2006; Mulenga et al., 2016; Thresh & Cooter, 2005). Studies conducted by Nyirahorana et al. (2017) in Rwanda showed that CMD symptom recognition can be improved by establishing demonstration plots for farmers. Furthermore, the implementation of good agricultural practices such as routine weeding of cassava farms will contribute to successful management of CMD since some of the weeds in farms can be alternative hosts for CMBs and perhaps sources of innoculum for whitefly spread of the virus.

In addition to CMD occurrence in the sampled cassava farms, high incidences of cassava green mite and cassava mealybugs were noted in several States in 2017. Studies have shown that mild mosaic symptoms are masked by leaf discolorations caused by green mites (Zinga et al., 2013), and mealybugs cause the characteristic bunching top symptom which makes CMD leaf distortion more severe (Parsa, Kondo, & Winotai, 2012). These may result in under‐ or over‐reporting of CMD incidence and symptom severity.

This study shows the presence of ACMV, EACMV and EACMCV in NC and SW Zones of Nigeria occurring as single or mixed infection in cassava and alternate host plants. ACMV was the predominant CMBs species found in both the NC and SW Zones of Nigeria as majority of CMD resulted from single ACMV infections. The predominance of single ACMV infection is similar to a previous country‐wide survey conducted in Nigeria (Alabi et al., 2008; Ariyo et al., 2005; Ogbe et al., 2006) and to other studies in West Africa (Pita et al., 2001; Torkpo, Offei, Danquah, & Gafni, 2017). In a previous study in Nigeria, Ogbe et al. (2006) observed that 74.1% of cassava samples collected had ACMV single infections which is similar to this study where 73.9% was observed. On the other hand, the distribution of single EACMV infection is often lower than that of ACMV (Fondong et al., 2000; Pita et al., 2001) as EACMV is more largely predominant in East African countries as compared to West Africa (Chikoti et al., 2013; Chikoti, Mulenga, Tembo, & Sseruwagi, 2019; Neuenschwander, Hughes, Ogbe, Ngatse, & Legg, 2002; Were et al., 2016).

Over 97% of the EACMV samples detected were found to be EACMCV which is no surprise given that Ogbe et al. (2003) already confirmed the high similarities between EACMCV and Nigerian EACMV isolates. In this study, the overall proportion of EACMV infections was low (<7%) which further points to the low prevalence of EACMV in Nigeria. The low occurrence of EACMV in Nigeria is probably because EACMV has not been present in Nigeria as long as ACMV (Ogbe et al., 2006). In this current study, single CMD infections caused by EACMV were observed in both Zones particularly in Ondo, Oyo and Benue States. This is, however, contrary to previous study by Ogbe et al. (2006) where EACMV single infection was only observed in Niger State. This implies that overtime if adequate management efforts are not implemented, EACMV may become even more widespread.

Besides the presence of single ACMV and EACMV infection, ACMV occurred in mixed infections with EACMV. This is similar to previous studies conducted across West Africa (Ariyo et al., 2005; Fondong et al., 2000; Ogbe, Thottappilly, Dixon, Atiri, & Mignouna, 2003; Pita et al., 2001) and East Africa (Chikoti et al., 2013; Harimalala et al., 2015; Were et al., 2016). The percentage of mixed infection recorded in this study (< 7%) was lower than the percentage found in previous country‐wide survey conducted in Nigeria in 1997–1998 (9.3%) and 2006 (24.1%) (Ogbe et al., 2006). Although the percentage of mixed infections seems to have reduced, the presence of mixed infection poses a risk of genetic recombination and has the potential to compound the problem if a more virulent CMB strain emerges (Berrie, Palmer, Rybicki, & Rey, 1998; Mulenga et al., 2016).

In addition to the potential of genetic recombination that can result from mixed infections, mixed infections also cause increased symptom expression in infected plants (Fondong et al., 2000). In previous studies conducted in Nigeria, plants with mixed CMB infection resulted in more severe symptoms than plants with single infection of either ACMV or EACMV infections (Ogbe et al., 2006). Similarly, in the current study, plants that had mixed ACMV and EACMV infections also had higher symptom severity scores as compared to plants that were infected with either ACMV or EACMV alone. Severe symptoms caused by mixed infections was also observed in Cameroon (Fondong et al., 2000), Cote d'Ivoire (Pita et al., 2001), Zambia (Chikoti et al., 2013), Kenya (Mwatuni et al., 2015), Tanzania and Uganda (Harrison, Zhou, Otim‐Nape, Liu, & Robinson, 1997). Increased symptom severity in mixed infection is attributed to the synergistic relationship between strains of CMBs involved in the mixed infection and an increase in plant virus titre (Naseem & Winter, 2016). It is, however, important to note while symptom expression is increased in mixed infection, it is also dependent on the virus strain infecting the plant and the variety of cassava planted (Ogbe et al., 2003).

In this study, approximately 35% of the asymptomatic cassava samples analysed were infected by at least one CMB. Plateau State (NC) recorded the lowest CMD incidence and was also one of the States with the highest proportion of ACMV infected plants. Symptom expression can be dependent on the type of cassava variety cultivated. Tolerant varieties such as TME 419 have the ability to repress CMD symptoms and as such reduce the effect of CMD on the tuber yield. A few of such tolerant varieties were recorded in this study. This underpins the importance of laboratory diagnosis during the selection of planting materials since selection and cultivation of such asymptomatic infected cassava plants will result in early onset of disease with resultant detrimental consequences on tuber yield; furthermore, such asymptomatic infected cassava plants may also act as reservoirs for the spread of CMD via the whitefly vector.

In addition to the presence of CMBs in asymptomatic samples, symptomatic samples that were unreactive to any of the primer pairs utilised in this study were also observed. This is similar to previous reports (Aloyce, Tairo, Sseruwagi, Rey, & Ndunguru, 2013; Chikoti et al., 2013; Harimalala et al., 2015; Mulenga et al., 2016; Ogbe et al., 2006; Zinga et al., 2013), where unidentified CMB was reported to have caused the CMD symptoms observed in cassava farms assessed. Further studies are needed for the detection of the virus(es) responsible for the symptoms observed in these plants.

## 5 CONCLUSION

This study provides an updated status of CMD incidence in cassava fields and the distribution of ACMV, EACMV and EACMCV in SW and NC Zones of Nigeria where nearly 50% of cassava production takes place in Nigeria. The study confirms that ACMV is still the predominant CMB strain in the region as EACMV occurred in low percentages in both Zones. The presence of CMB in asymptomatic samples further buttresses the need for a functional clean seed certification system in Nigeria. Furthermore, the presence of symptomatic samples that were unreactive to primer pairs utilised in this study highlights the need for further study to determine the etiology of the CMB involved.

## CONFLICT OF INTEREST

The authors declare that they have no known conflict of interest.
